# Analyzing the association between menstrual coitus and endometriosis’ pathogenesis: A narrative review

**DOI:** 10.1177/17455057241305072

**Published:** 2025-02-20

**Authors:** Giulia Emily Cetera, Maria Carmen Punzi, Camilla Erminia Maria Merli, Paolo Vercellini

**Affiliations:** 1 Gynecology Unit, Fondazione IRCCS Ca’ Granda Ospedale Maggiore Policlinico, Milan, Italy; 2 Department of Clinical Sciences and Community Health, Academic Center for Research on Adenomyosis and Endometriosis, Università degli Studi di Milano, Milan, Italy; 3 Business-Society Management Department, Rotterdam School of Management, Erasmus University Rotterdam, Rotterdam, the Netherlands

**Keywords:** endometriosis, menstruation, menstrual coitus

## Abstract

Across studies, the percentage of individuals reporting regularly engaging in menstrual coitus ranges between 4% and 43%. Although no clinical guideline recommends avoiding sexual activity during menstruation, according to some researchers such practice may favor both retrograde menstruation and sexually transmitted diseases, two phenomena that are thought to play a role in endometriosis’ pathogenesis. Given this background, we analyzed the existing evidence regarding the association between menstrual coitus and the prevalence of endometriosis by conducting a PubMed database search on February 15, 2024. We considered all original, full-length studies written in English. Results were conflicting. When interviewing 489 infertile women, Filer and Wu found that the frequency of surgically diagnosed endometriosis was significantly higher among those engaging in menstrual coitus (17.5% versus 10.9%; *p* < 0.05). In their case-control study on 555 women with (*n* = 185) and without (*n* = 370) endometriosis, Mollazadeh and co-workers confirmed an increased risk of endometriosis among those engaging in menstrual coitus compared to those who did not (OR 5.23; 95% CI 2.16–12.66). However, in Meaddough and colleagues’ retrospective case-control study on 2012 women, with (*n* = 1517) and without (*n* = 495) endometriosis, menstrual coitus was significantly less frequent in women with endometriosis compared to controls (27% versus 35%; *p* = 0.002). Treloar and co-workers also failed to prove such an association. The evidence available at the present moment is insufficient to confirm the hypothesis that menstrual coitus plays a role in the pathogenesis of endometriosis.

## Introduction

Endometriosis is characterized by the growth of tissue resembling the endometrium outside the uterine cavity. This condition is often accompanied by pelvic adhesions and localized inflammatory responses. Although no single theory can fully explain all cases of endometriosis, increasing evidence points to a significant role of disrupted immune homeostasis in the peritoneal environment, leading to a pro-inflammatory state. Factors that increase retrograde menstruation and the inability of the immune system to clear endometriotic cells shed into the peritoneum are among the theories that have been emphasized in recent years.^1,2^

First proposed by Sampson in 1924, the retrograde menstruation theory is based on the hypothesis that menstrual blood refluxes from the endometrial cavity into the abdominal cavity during each menstrual period, enabling endometrial cells to implant onto abdominopelvic structures.^3^ Emerging evidence suggests that genital and gut microbiota imbalance may also be involved in the pathogenesis of endometriosis.^4,5^ Microbes may modulate immune responses,^6^ influence estrogen metabolism,^7^ affect circulating estrogen concentrations,^8^ and induce phenotypic changes in endometrial cells.^9^ Moreover, chronic endometritis could alter uterine contractility, potentially increasing retrograde menstruation.^10^

Historically, intercourse during menstruation has been—more or less overtly—condemned in many societies. Reasons for such proscription have been religious, cultural, hygienic, or aesthetic.^11–16^ Some individuals avoid sex while bleeding for fear of the transmission of infectious diseases. Others (both women and men) consider menstruation something to hide and keep secret.^15,17^ This latter belief is rooted in the taboo of menstruation, which is as old as humankind^8^ and is still largely prevalent in most societies, perpetuated by the marketing of mainstream menstrual products, which emphasize the need for secrecy and freshness through the use of euphemisms and delicate, “feminine” imagery.^18–^20^^ For such reasons, many consider sexual activity during menstrual activity as awkward, dirty, and disgusting.^13,21^ Physical pain related to dysmenorrhea and discomfort related to bloating may be further deterrents to engaging in sex at this time, although the messiness of menstrual coitus also seems to play a role.^13^

As a result, independent from age and ethnicity, women have sex more often while not menstruating than while on their periods.^17,22,23^ Orgasm rates also seem to be significantly less frequent during menstruation, both in women and in men, although masturbation rates do not seem to change.^24^ However, menstrual coitus is more frequent than one might believe. Across studies, the percentage of individuals reporting ever having had sex during menstruation varies between 50% and 66%; while the percentage of those regularly engaging in menstrual coitus ranges between 4% and 43%. Factors associated with menstrual coitus include Caucasian or Hispanic ethnicity, higher education, being aroused by atypical sexual activities, being in a relationship, having had a greater number of lifetime partners and having greater coital frequency.^13,17,23,25–29^ When considering adolescents, menstrual coitus seems to be more frequent among those of younger age, probably due to the fact they have fewer occasions to engage in sexual intercourse.^17^ Recent years have also seen an increase in online articles on women’s magazines and “pop-health” websites that point to orgasm as a “natural” pain relief option, although no peer-review studies to date have proven such correlation. Media attention on the topic sparked when in 2021, sex toy company Womanizer and menstrual cup company Lunette partnered on an experimental, non-peer-reviewed study of 486 people, the “menstrubation study,” which showed indication of a potential of masturbation for menstrual symptoms relief, including cramps.^30^

Although no clinical guideline recommends avoiding sexual activity during menstruation, researchers’ opinions in this regard are not unanimous. According to some, menstrual coitus and orgasm during menses may favor retrograde menstruation and as such may play a role in endometriosis’ pathogenesis.^25,28,31^ According to others, sexual activity while bleeding may be a risk factor for sexually transmitted diseases and pelvic inflammatory disease.^26,32^ Interestingly, the emerging theory according to which genital microbiota imbalance could play a role in endometriosis’ pathogenesis^9^ may represent a link between infectious diseases and endometriosis, further supporting the hypothesis that menstrual coitus and endometriosis are related.

Given this background, the aim of our narrative review is to analyze the existing evidence regarding the association between sexual activity during menstruation and endometriosis’ pathogenesis.

## Methods

We considered all original, full-length studies on the association between menstrual coitus (or non-coital sexual activities leading to orgasm, performed during menses) and the prevalence of endometriosis. Articles were included if written in English and published in peer-reviewed journals. Articles regarding the implications of endometriosis on menstrual coitus and on specific populations in which menstrual coitus is forbidden were excluded. No limit to the year of publication or to patients’ age was applied. Abstracts, conference papers, and articles not reporting original data were also excluded.

Studies were identified by a PubMed database search that was conducted on February 15, 2024, using the keyword “endometriosis” in combination with “menstrual coitus,” “intercourse,” “sex,” “sexual,” “orgasm,” “menstrual,” “menstruation,” “period,” “menses,” “bleeding,” “blood,” and “prevalence.” We screened references from relevant publications and searched for further articles using PubMed’s “Similar articles” and “Cited by” functions.

Studies defining the retrograde menstruation theory and the genital microbiota theory were also included to describe the etiological theories of endometriosis. Lastly, epidemiological studies analyzing the prevalence of genital tract infections in women engaging or not in sexual activities during menses were included to further investigate the possible link between menstrual coitus, infections, and endometriosis.

## Results

### Studies on the association between menstrual coitus and endometriosis

It has been suggested that the uterine contractions occurring during female orgasm may augment intrauterine pressure, leading to an increase in the quantity of blood and endometrial cells spilling out of the fallopian tubes during menses.^11,28^ Also, the risk of contracting sexually transmitted infections appears to be increased during menses,^12,33,34^ potentially favoring the onset of endometriosis if the microbiome theory were true. Accordingly, various authors have observed an increased prevalence of endometriosis among women engaging in menstrual coitus, although others have failed to find such an association (Table 1).

**Table 1.** Studies analyzing the association between endometriosis and menstrual coitus.

Author (year)	Country	Study type	Study population	Methods	Main results
Filer and Wu (1989)^25^	United States	Observational study	489 infertile women, with (*n* = 71) or without (*n* = 418) surgically-diagnosed endometriosis	Interview (not reported if oral or written)	Endometriosis was more frequent among those having menstrual coitus (17.5% versus 10.9%; *p* < 0.05)
Mollazadeh et al. (2019)^28^	Iran	Case-control study	185 women with a histological diagnosis of endometriosis and 370 women self-reporting not having endometriosis	Questionnaire	The risk of endometriosis was higher in those engaging in menstrual coitus (OR 5.23; 95% CI 2.16–12.66) and in non-coital activities leading to orgasm (except for anal sex), (OR 2.9; 95% CI 1.28–6.55), compared to those who did not
Meaddough et al. (2002)^29^	United States	Case-control study	1517 women with a self-reported diagnosis of endometriosis and 495 controls reporting not having endometriosis	Written survey	Both menstrual coitus and non-coital sexual behavior leading to orgasm were lower in women with endometriosis compared to women without the condition (27% versus 35% for menstrual coitus; *p* = 0.002; 23% versus 31% for non-coital activity; *p* = 0.001)
Treloar et al. (2010)^35^	Australia	Case-control study	268 women with surgically-confirmed endometriosis and 244 women without endometriosis	Self-administered questionnaire	Sexual intercourse during menstruation was not associated with endometriosis

Back in 1989, Filer and Wu interviewed 489 infertile women on their sexual habits and found that the frequency of surgically diagnosed endometriosis was significantly higher among those who reported having menstrual coitus, compared to those who reported not having sex during menses (17.5% versus 10.9%; *p* < 0.05). Moreover, the prevalence of endometriosis was higher among those having frequent menstrual coitus, compared to those occasionally engaging in such a practice, although the difference was not significant.^25^ Mollazadeh and colleagues confirmed such association between menstrual coitus and endometriosis. In their case-control study on 185 women with a histological diagnosis of endometriosis and 370 women referred for a gynecological evaluation for vaginitis or an annual check-up, the risk of endometriosis was estimated to be approximately five times higher in those stating they had menstrual coitus compared to those who did not (OR 5.23; 95% CI 2.16–12.66). Moreover, the percentage of women reporting they always had menstrual coitus was significantly higher among those with endometriosis compared to controls (20% versus 3.6%, *p* < 0.001). The risk of endometriosis was higher also among those engaging in non-coital sexual activities leading to orgasm during menstruation (except for anal sex), compared to those who did not (OR 2.9; 95% CI 1.28–6.55). Differences among cases and controls remained significant even after controlling for the effect of possible confounders such as socio-demographic variables, menstrual characteristics, medical and gynecological history, and symptoms including dysmenorrhea and dyspareunia.^28^

Two studies failed to find a positive association between menstrual coitus and endometriosis. In Meaddough and co-workers’ retrospective case-control study on 2012 women, with (*n* = 1517) or without (*n* = 495) endometriosis, both menstrual coitus and non-coital sexual behavior leading to orgasm resulted to be significantly less frequent in women with endometriosis compared to women without the condition (27% of women with endometriosis performing menstrual coitus versus 35% of women without endometriosis performing menstrual coitus; *p* = 0.002; 23% of women with endometriosis having non-coital sexual activity during menses versus 31% of women without endometriosis having non-coital sexual activity during menses; *p* = 0.001).^29^ However, the quality of these results may be limited by the fact that the diagnosis of endometriosis was self-reported and not based on a clinical evaluation, and that confounding variables such as dyspareunia were not included in the analysis. It may be argued that women with endometriosis may have not been engaging in sexual activities during menses due to an increase in their painful symptoms at such times. Lastly, in Treloar and colleagues’ case-control study on 268 women with a surgical diagnosis of endometriosis and 244 women without the disease, sexual intercourse during menstruation was not associated with endometriosis.^35^

Although they did not specifically analyze the association between menstrual coitus and endometriosis, Cutler and co-workers observed that among 160 peri-menopausal women with a mean age of 48.8 years, those engaging in sex during menses were four times more likely to report heavier menstrual flows, compared to those abstaining from menstrual coitus (65.5% versus 14.8%). No significant difference was observed in coital frequency or in orgasm rates between those engaging in menstrual coitus and those who refrained from doing so. The authors suggest that the increase in menstrual flow reported by women having sex during menses could be due to increased uterine contractions, which could potentially favor retrograde menstruation, thus leading to the development of endometriosis.^36^ Although the study was limited by a series of drawbacks, first and foremost by the fact that the quantity of blood flow was not measured, but self-reported, Cutler’s results indirectly support the hypothesis of an association between menstrual coitus, retrograde menstruation, and endometriosis. Moreover, although recruited women were supposedly healthy, reported heavy menstrual bleeding could have been an indirect sign of undiagnosed adenomyosis.^36^

### Studies on the association between menstrual coitus and retrograde menstruation

At the present moment, no direct evidence of a positive association between menstrual coitus and retrograde menstruation is available.

### Studies on the association between menstrual coitus and infectious diseases

Multiple hypotheses have been raised to explain why the risk of sexually transmitted infections is increased during menses. Microorganisms appear to access the uterine cavity with greater facility due to an increased penetrability of the cervical mucous at this time, may feed on the iron contained in menstrual flow, and may easily reflux into the fallopian tubes together with menstrual blood during menstrual uterine contractions. This may determine the spread of pathogens in the peritoneal cavity, contributing to the development of pelvic inflammatory disease.^33,34^ Also, the vaginal microbiome and local immunity may undergo cyclic variations, which may result in a hampered antibacterial, antifungal, and antiviral protection during menstruation.^12^ This appears to be particularly true among women not using hormonal contraceptives.^37^ For example, at time of menstruation anti-gonococcal complement function is lowest^38,39^ and toll-like receptors are expressed less.^40^ According to some, menstrual-related modifications in the genital environment may also modify microrganisms’ virulence.^41^ As such, menstruation may represent a window of opportunity for pathogens to ascend into the upper-genital tract, causing infection.

Evidence regarding the association between menstrual coitus and the risk of sexually transmitted diseases is conflicting. McLaughlin and Griffiss reported a significantly increased risk of acquiring a Neisseria Gonorrhoeae cervical infection among women engaging in sexual intercourse during menses (OR 12.5, *p* = 0.05)^32^ and Tanfer and Aral observed that the cumulative incidence of self-reported sexually-transmitted diseases was greater among those who had sex during menses (OR 2.11, *p* < 0.001), even after controlling for the number of lifetime partners and for frequency of sexual intercourse.^26^ However, such association between menstrual coitus and increased risk of pelvic inflammatory disease failed to be proven in two studies.^25,42^ Curiously, Eschenbach and colleagues demonstrated a higher frequency of pelvic inflammatory disease in women avoiding menstrual coitus (percentages and significancy not reported in the original article),^42^ while Filer and Wu failed to find a significant association between the two variables.^25^

## Discussion

Although evidence in this regard is scanty and conflicting, menstrual coitus and non-coital activities leading to orgasm performed during menstruation could be associated with endometriosis.^25,28^

Among all, two pathogenic theories of endometriosis could support the role of menstrual coitus in the development of the disease: the retrograde menstruation theory and the microbiome theory. According to the former, the menstrual blood that refluxes from the endometrial cavity into the abdominal cavity during each menstrual period would determine the implant of endometrial cells onto abdominopelvic structures.^3^ By determining an increase in myometrial contractions, menstrual coitus and female orgasm during menses may favor such reflux of endometrial blood into the peritoneum. If this were true, menstrual coitus could explain the discrepancy between the 10% prevalence of endometriosis among women of reproductive age and the fact that menstrual blood is present in the peritoneal cavity of 90% of healthy women with patent fallopian tubes.^43^

According to the microbiome theory, endometriosis could be the consequence of an imbalance in the genital microbiota, which may modify the ability of endometrial fibroblasts to proliferate, migrate, and adhere to the abdominopelvic structures.^3,4^ Moreover, microbial organisms may interfere with estrogen metabolism, while chronic endometritis could augment uterine contractility, determining an increase in the volume of the endometrial blood which refluxes in the peritoneum.^12^ As menstruation probably represents a window of opportunity for pathogens ascending into the upper-genital tract,^12^ engaging in sexual activity at such time could further disrupt the genital microbiome and facilitate the genesis of genital infections, ultimately increasing the risk of developing endometriosis. This theory is supported by the evidence that endometriosis is associated with an increased frequency of genital tract infections.^9,44–48^ This said, infections could be a consequence of other conditions associated with endometriosis, including low-grade pelvic inflammation, menorrhagia, changes in immunology, and hormonal therapy.^5^

However, a causative relation between sexual activity during menses and endometriosis is yet to be proven. An association between the two has been reported in two studies,^25,28^ which also report a greater prevalence of endometriosis among those frequently engaging in menstrual coitus, compared to those engaging in sexual activity only occasionally during menses. Yet, the difference was statistically significant only in one study.^28^ In this study endometriosis was also associated with non-coital sex leading to female orgasm. It could be speculated, in fact, that regardless of vaginal penetration, uterine contractions due to female orgasm increase the volume of blood spilling through the Fallopian tubes into the peritoneal cavity. Two further studies failed to prove such a positive association.^29,35^ In fact, contrary to Mollazadeh and co-workers., Meaddough and colleagues hypothesized that sexual activity during menses may actually facilitate anterograde blood flow through the cervix.^29^ However, the results of the latter study may have been influenced by the fact that endometriosis diagnoses were self-reported and that confounding variables such as dyspareunia were not analyzed.

Further studies considering confounding variables such as the presence of painful symptoms at time of menses, the frequency of menstrual coitus, a history of tubal ligation, the use of condoms, and the timing of the onset of endometriosis’ symptoms with respect to that of when the women started engaging in sexual activities during menses (temporality) are needed. In fact, the absence of high-quality studies and the exiguous number of those existing are the main limitations of this review, which as a result can not draw any conclusions regarding the role of menstrual coitus and endometriosis. However, we believe that having shed light on the necessity of further research on this topic is the main strength of our work.

In conclusion, women should be informed that, apart from cultural, religious, or personal beliefs, reliable recommendations in favor or against engaging in sexual activity during menstruation can not be currently provided.

## Conclusions

Although menstrual coitus and non-coital sexual activities are discouraged in many cultures and religions, a scientific explanation for such an interdiction is yet to be found. In fact, study results are conflicting and the possible mechanisms behind the association between menstrual coitus and endometriosis are yet to be clarified. Long follow-up prospective studies on endometriosis patients, as well as retrospective studies based on large samples of women with a histological diagnosis of endometriosis, are needed. Also, attention to the type and frequency of sexual activities conducted during menses, as well as to temporality (date of endometriosis diagnosis compared to date of menarche, date of coitarche and that in which the woman started engaging in menstrual coitus) are fundamental to establish causative relations.

## Declarations

**Ethics approval and consent to participate:** Not applicable as the study is based exclusively on published literature.

**Consent for publication:** Not applicable.

**Availability of data and materials:** Not applicable.
